# Editorial: *Listeria monocytogenes*: do we know enough about this pathogen?

**DOI:** 10.3389/fmicb.2025.1698291

**Published:** 2025-09-25

**Authors:** Kathrin Rychli, Natalia Wiktorczyk-Kapischke, Krzysztof Skowron

**Affiliations:** ^1^Centre for Food Science and Veterinary Public Health, University of Veterinary Medicine Vienna, Vienna, Austria; ^2^Department of Microbiology, Ludwik Rydygier Collegium Medicum in Bydgoszcz, Nicolaus Copernicus University in Toruń, Bydgoszcz, Poland

**Keywords:** whole genome sequencing (WGS), transcriptome sequencing, genetic diversity, biofilm, food safety

*Listeria* (*L*.) *monocytogenes* is a gram positive foodborne pathogen responsible for listeriosis, a rare, but severe infection disease. *L. monocytogenes* was discovered by E.G.D. Murray in 1926 investigating an outbreak affecting rabbits and guinea pigs in animal care houses in England (Murray et al., 1926). First human cases were reported in 1929, considering listeriosis as a zoonosis. More than 40 years later, a human listeriosis outbreak was finally directly linked to the consumption of *L. monocytogenes* contaminated food (Schlech et al., 1983). Now, *L. monocytogenes* is unambiguously recognized as a food borne pathogen, that can infect humans and animals. In healthy individuals, listeriosis is presented as a non-invasive, self-limiting gastroenteritis, but *L. monocytogenes* can also be silently present in the gastro-intestinal tract. In contrast, in immunocompromised and elderly individuals, newborns and pregnant women, a severe and systemic infection can occur, resulting in meningoencephalitis, septicaemia or abortion (Vazquez-Boland et al., 2001).

Our knowledge on the occurrence, genetic diversity, pathogenicity and behavior of *L. monocytogenes* has largely increased in the last decades using whole genome sequencing and analysis, transcriptomics and *in vitro* and *in vivo* virulence models. Moreover, *Listeria* is widely used as a model microorganism studying the interplay between a pathogenic microbe, host tissues and microbiota *in vivo*.

But there are still many open questions. We still do not understand the behavior of *Listeria* on food and within the food producing environment particularly in relation to microbial interactions and biofilm formation. Moreover, our knowledge on the genetic diversity of *L*. *monocytogenes* from “non-classical- sources” like soil or unexplored countries is still limited.

This Research Topic, which is composed of nine articles, addresses these aspects including different environments like soil of carrot farming, leafy vegetables, beef, drains in a meat processing plant and a frozen vegetable producing environment.

Nowak et al. analyzed the prevalence of *L. monocytogenes* in soil samples from organic carrot crops in Poland. They found *L. monocytogenes* in 10.8% of the samples. The isolated strains were characterized including antibiotic resistance, disinfectant tolerance, biofilm formation and virulence.

Virulence of *L. monocytogens* was also the focus of the review by Sousa et al.. They in depth discussed the different methods available to evaluate virulence among clonal complexes.

Prieto et al. focused on the genetic diversity of *L. monocytogenes* in the food chain in Montenegro using whole genome sequencing and analysis. The 160 isolates belonged to 21 clonal complexes, among them ST8, ST9, ST121 and ST155 were the most prevalent. This was the first study of *L. monocytogenes* from Montenegro.

The study of Zhang et al. also studied the genetic diversity of *L. monocytogenes*, but they focused on one food source namely beef. They conducted gene profiling of virulence and stress resistant genes and pangenomic analysis including international 344 strains.

The study of Fagerlund et al. investigated the temporal variation and population dynamics of *L. monocytogenes* in drains in a meat processing plant in Norway, focusing on the diversity of *L*. *monocytogenes* and the impact of the resident microbiota. *L. monocytogenes* was detected in the majority of samples and four different CCs were identified with up to three CCs in the same sample. Analysis of the microbiota in drains and enrichment cultures by 16S rRNA gene amplicon sequencing and metagenomic or quasimetagenomic sequencing, revealed that the drain microbiota remained relatively stable over time, with *Pseudomonas, Acinetobacter, Janthinobacterium, Chryseobacterium, Staphylococcus*, and *Sphingomonas* as the most commonly identified genera. There were no apparent differences in the microbial genera present in *L. monocytogenes* positive and negative drains or samples.

Pracser et al. analyzed the occurrence of *Listeria* in biofilms in a European frozen vegetable processing facility. Biofilms were present on 12.7% sites. In two cases, *L. innocua* was detected in a biofilm, which was the first study confirming the presence of *Listeria* within a biofilm in a real environment. Furthermore, the resident microbial and the co-occurrence of bacterial taxa with *Listeria* were investigated by 16S rRNA gene sequencing. *Pseudomonas, Acinetobacter*, and *Exiguobacterium* dominated the microbial community of the processing environment. Using differential abundance analysis, Enterobacterales and *Carnobacterium* were found to be significantly higher abundant in *Listeria*-positive samples.

Culliney and Schmalenberger aimed to analyse the bacterial community of leafy vegetables like spinach and their effect on *L. monocytogenes* growth post-harvest. They further tested the effect of the different cultivation conditions like polytunnel on the microbiota and revealed that cultivation conditions determine bacterial phyllosphere community structure, which consequently influenced *L*. *monocytogenes* growth.

As exopolysaccharides enhance the ability of *L. monocytogenes* to colonize and persist on surface of fresh fruit and vegetables, Elbakush et al. investigated the effect of maple compounds on biofilm formation. They discovered that maple lignans inhibit not only biofilm formation, but enhanced biofilm dispersal via sortase A inhibition. The role of sortase A is to anchors surface proteins to the cell wall, including EPS.

How exogenous fatty acids (FA) influence the growth of *L. monocytogenes* at low temperature was the research question of Quilleré et al. using transcriptomic analysis. They demonstrated that *Listeria* regulates the synthesis of saturated FA in its membrane. Moreover, they detected that unsaturated FA upregulated genes involved in flagellar assembly, resulting in numerous and long-looped flagella.

Summarizing, this Research Topic enlarges our knowledge on the interaction of *L. monocytogenes* with bacteria in food and food producing environment. Furthermore, it unravels genetic diversity of *L. monocytogenes* strains from novel sources.
